# Uptake of ammonia by ice surfaces at atmospheric temperatures†

**DOI:** 10.1039/d4fd00169a

**Published:** 2024-11-16

**Authors:** Clemens Richter, Shirin Gholami, Yanisha Manoharan, Tillmann Buttersack, Luca Longetti, Luca Artiglia, Markus Ammann, Thorsten Bartels-Rausch, Hendrik Bluhm

**Affiliations:** a Fritz Haber Institute of the Max Planck Society Faradayweg 4–6 14195 Berlin Germany bluhm@fhi-berlin.mpg.de crichter@fhi-berlin.mpg.de; b PSI Center for Energy and Environmental Sciences, Paul Scherrer Institute CH-5232 Villigen PSI Switzerland thorsten.bartels-rausch@psi.ch

## Abstract

We present an ambient pressure X-ray photoelectron spectroscopy investigation of the adsorption of ammonia on ice over the temperature range −23 °C to −50 °C. Previous flow tube studies have shown significant uptake of ammonia to ice at these temperatures, which was linked to the incorporation of ammonium into the ice crystal lattice. Our present investigation shows a significant uptake of ammonia to the ice interface, with ammonia concentrations exceeding those measured in past studies for the case of bulk snow and ice. We also have indication that some of the ammonia is protonated at the ice surface and thus adsorbed there as ammonium ions. The impact of high ammonia concentrations at the air–ice interface on the surface chemistry of ice clouds is discussed. The present study lays the groundwork for investigating the reaction of adsorbed ammonia with other trace gases in the atmosphere, which is demonstrated with the example of a proof-of-principle experiment of ammonia’s interaction with acetic acid.

## Abstract 2

We present an ambient pressure X-ray photoelectron spectroscopy investigation of the adsorption of ammonia on ice over the temperature range −23 °C to −50 °C.

## Graphical abstract

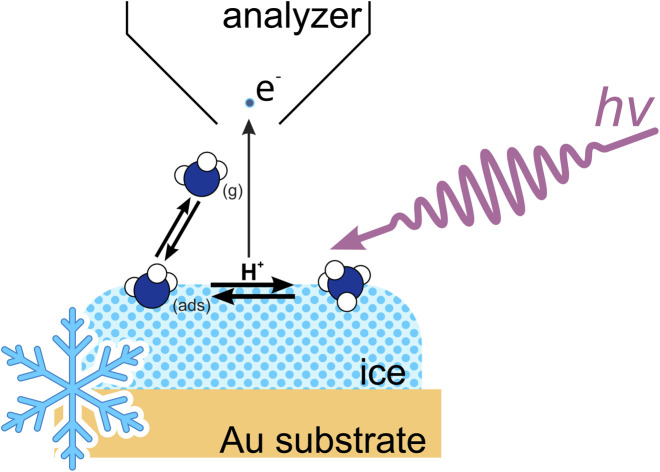

## 1 Introduction

Ammonia (NH_3_) plays a central role in determining the pH value of atmospheric cloud droplets and aerosol particles.^1–3^ It is the most abundant alkaline trace gas in the troposphere^4^ with typical atmospheric concentrations in the sub-ppb to tens of ppb range.^5^ The interaction of NH_3_ with acidic trace gases is a key mechanism for the nucleation and formation of secondary aerosols in the atmosphere.^6^ This reaction leads to the formation of ammonium (NH_4_^+^) species, a major inorganic aerosol component worldwide.^7^ It has also been detected, for instance, in Antarctic coastal snow, after long-range transport and wet precipitation.^8^ Abbatt *et al.*^9^ and Wentworth *et al.*^10^ suggest that the bidirectional NH_3_ exchange between the atmosphere and the land–ocean surface is significant and needs to be included in chemical transport models. This is demonstrated by the fact that NH_3_ was detected in the Arctic^9^ as well as in the upper troposphere,^11^ where cirrus clouds are well known to adsorb acidic trace gases and thus impact their atmospheric budget.^12^

Despite its abundance and importance in atmospheric multiphase processes and reactions, the acid–base chemistry of NH_3_ in contact with ice and snow under conditions relevant to the Earth’s cryosphere has so far not been studied in detail. This is an important gap in our knowledge in view of the rising NH_3_ concentrations in the atmosphere, in particular over the last decade. The concentration of NH_3_ in the atmosphere is expected to continue to rise due to, *e.g.*, the increased use of nitrogen-containing fertilizers. This development shifts the composition of atmospheric reactive nitrogen from oxidized nitrogen compounds toward a greater prevalence of reduced nitrogen compounds like NH_3_.^13,14^

It is well known that in aqueous environments NH_3_ can undergo protonation to form NH_4_^+^:NH_3_ + H_2_O ⇌ NH_4_^+^ + OH^−^

Our experiments address the question of whether NH_3_ adsorbs molecularly on the ice at arctic temperatures (−23 to −52 °C) or whether it undergoes protonation to a significant degree.

Some earlier investigations have addressed the adsorption state of NH_3_ on ice, albeit at temperatures well below those in the Arctic or the upper parts of the troposphere. The study by Ogasawara *et al.* indicated a rapid protonation of NH_3_ when the ice substrate was heated from −235 °C to −153 °C,^15^ a result that was also supported by Monte Carlo simulations.^16^ A subsequent investigation by Lee and Kang^17^ was carried out at higher temperatures (around −70 °C), but did not show any indication for the protonation of NH_3_, which would have been expected if protonation is observed already at lower temperatures. Lee and Kang argued that the protonation observed in the previous investigation was driven by incomplete wetting of the ice film on the metal substrate and protonation of NH_3_ was governed by the interaction with the metal substrate in the presence of water molecules that foster proton transfer. They also showed that at temperatures of around −70 °C incorporation of NH_3_ into the ice bulk was negligible.

One major pathway for the trapping of atmospheric trace gases is their incorporation into the bulk of growing ice particles in clouds. Hoog *et al.*^18^ argued that ammonia is efficiently trapped by growing ice due to the high solubility of NH_4_^+^ in water. Indeed, NH_4_^+^ and NH_3_ are generally thought to have a high solubility in ice (about 2 g l^−1^) due to the ability of NH_4_^+^ to substitute for water molecules in the ice lattice.^19^ However, it was pointed out that these measurements are difficult and prone to large uncertainties.^19–21^ Kärcher *et al.* proposed that trapping of trace gases in ice is governed by their adsorption at the ice surface and subsequent diffusion into the bulk, a process that is also influenced by the growth rate of the ice crystal.^22,23^ Incorporation into bulk ice thus provides a pathway for the uptake of very high amounts of trace gases, compared to trapping mechanisms based on purely surface adsorption. For the case of ammonia this was shown by Hoog and coworkers^18^ who studied the uptake of NH_3_ to ice crystals at temperatures above −20 °C and NH_3_ gas phase concentrations of up to 10 ppm and found that NH_3_ enters the ice phase as NH_4_^+^, which is then incorporated into the ice lattice.

**Fig. 1:**
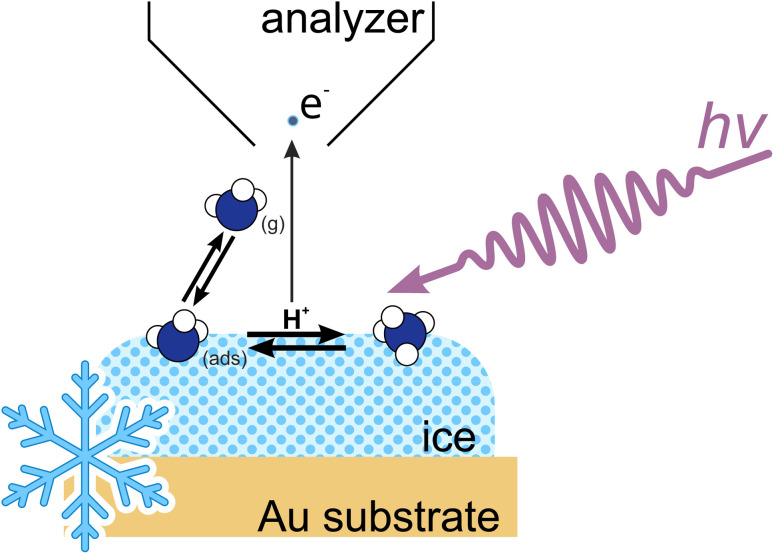
Schematic of NH_3_ adsorption at the ice–vapor interface in our experimental setup.

In addition to the bulk, the interface layer (the first few nanometers) on ice also has a potentially high capacity to capture trace gases, as seen for strong acids such as HCl and HNO_3_.^24,25^ To observe this phenomenon, interface-sensitive techniques are required to directly determine the concentration of adsorbed species and to evaluate the impact of surface processes on the trapping of trace gases at the ice–air interface. Our present study thus focuses on the adsorption of ammonia on ice, which we investigate with ambient pressure X-ray photoelectron spectroscopy (APXPS) at temperatures relevant to polar regions and the upper parts of the troposphere (see Fig. 1).

We find evidence that under these conditions NH_3_ is present at the air–ice interface. We also present a proof-of-principle investigation of the heterogeneous reaction of adsorbed NH_3_/NH_4_^+^ with a relevant trace gas in the atmosphere – acetic acid. The feasibility of APXPS studies of these phenomena paves the way for in-depth investigations of heterogeneous reactions (*e.g.* the chemical nature of adsorbates and reaction products) on ice surfaces taking place in polar regions and on frozen aqueous aerosol particles occurring in the troposphere.

## 2 Experimental

The experiments were performed at the X07DB *in situ* spectroscopy beamline of the Swiss Light Source (SLS) of the Paul Scherrer Institute using the Ambient Pressure Photoemission endstation.^26^ The endstation consists of a differentially pumped hemispherical electron analyzer (Scienta R4000 HiPP-2) attached to a reactor cell with a temperature controlled sample holder. Connected to the reactor cell is a gas dosing system which controls the partial pressures of the trace gases and water vapor. All measurements were performed at partial pressures of water up to 2.5 mbar to maintain the prepared ice samples in equilibrium with their respective vapor pressure corresponding to the ice temperature. Photon energies were chosen to ensure that spectra from all relevant core-levels were obtained at similar photoelectron kinetic energies of about 245 eV.

### 2.1 Sample preparation

Ice samples were grown on a Au-coated sample holder positioned in the reactor cell at a distance of several millimeters from the differentially-pumped entrance aperture of the electron analyzer. In this position any influence of the reduced water vapor pressure right in front of the aperture is avoided during ice growth. Water vapor was dosed into the reactor cell at a partial pressure slightly exceeding that of the equilibrium vapor pressure at the desired ice temperature to establish a slight oversaturation. Subsequently, the temperature of the sample holder was reduced until the formation of ice nuclei was observed by eye. The formation of ice was also indicated by a decrease of the water vapor pressure in the reactor cell. Once ice nucleation was established the ice was allowed to grow slowly for about 1 hour, until a closed polycrystalline ice film of a few hundred micrometer thickness was formed.

XPS measurements on these ice films were typically performed in an additional flow of Ar in the experimental cell at partial pressures of about 0.2 mbar to 0.4 mbar. This background gas helped to minimize perturbations of the ice due to radiative heating from the reactor cell walls and the aperture of the electron analyzer.^27^ In addition, the use of a background gas partially compensates the charging of the insulating ice film due to electron emission and offers the possibility to vary the gas phase composition in the experimental cell at a constant total pressure (*p*_cell_ = constant on the order of 0.4 to 2.5 mbar). NH_3(g)_ was dosed onto the ice films from a premixed gas mixture of 3% NH_3_ in He. In that manner we were able to dose NH_3_ at partial pressures between 1.2 × 10^−3^ mbar to 6.0 × 10^−3^ mbar (1.2 to 6 ppm).

### 2.2 Phototelectron spectroscopy

For each experimental run a set of X-ray photoemission spectra was first taken of the as-prepared ice film at the temperature of interest, and then during the exposure of the ice surface to NH_3_. We also followed the evolution of the surface chemical composition once the NH_3_ flow into the reactor cell was stopped. Typical durations for a single experiment varied from 2 to more than 5 hours. O 1s and N 1s spectra were taken at photon energies of 780 eV and 650 eV, respectively, to ensure that the photoelectrons from the different core levels have a comparable kinetic energy and thus comparable electron probing depths, which is about 1.7 nm for electrons with a kinetic energy of 250 eV detected at an angle of 30° relative to the surface normal.^28^

The spectra were fitted using the KolXPD software package (Kolibrik.net, Czech Republic). For all spectra a linear background was subtracted, and Gaussian peaks were used to fit components due to substrate and adsorbate species. Peaks due to gas phase species were fitted using Voigt profiles. Example spectra and a more detailed description of the fitting routine and constraints are shown in the ESI (see Fig. S4–S7).†

## 3 Results and discussion

In the following, we describe the results of the XPS experiments on the uptake of NH_3_ by ice surfaces. We start with the description of the principal components of the N 1s spectra of as-grown and ammonia-covered ice surfaces before describing the uptake experiments.

### 3.1 Principal components of the N 1s spectra

**Fig. 2:**
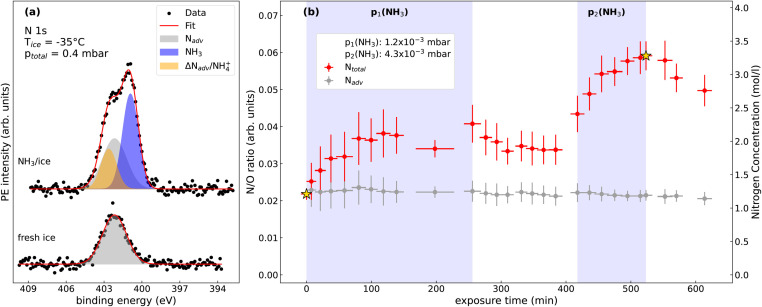
(a) N 1s spectra of freshly prepared ice (bottom) and NH_3_/ice (top) at −35 °C at a partial pressure *p*(NH_3_) of 4.3 × 10^−3^ mbar. The spectra were scaled to the same background intensity. Subsequently, the linear background was subtracted. (b) N/O ratio and estimated concentrations of N_total_ (red) and N_adv_ (grey) as a function of exposure time. Star symbols indicate the data corresponding to spectra shown in (a). Uncertainties on the *x*-axis stem from the acquisition times required for a set of N 1s and O 1s spectra. Uncertainties in the *y*-direction have been evaluated as the standard deviation (1*σ*) from the signal-to-noise ratios of the core-level spectra.

Fig. 2(a) shows a representative N 1s spectrum of a freshly prepared ice sample (bottom trace), and that for NH_3_ adsorbed on the same ice sample during exposure to NH_3(g)_ when a steady-state of surface adsorption at an ice temperature of −35 °C was reached (top trace). The freshly-grown ice sample already shows a significant contribution of a nitrogen species (grey-shaded peak), which is due to adventitious contamination, which was present in all prepared ice samples. The precise nature of this species and its origin could not be unambiguously determined, though it is likely due to residual contamination of the reactor cell, which, despite our best efforts, could not be removed. As we will show in the following, this species has only a marginal effect on the results of the investigation since it behaves like a bystander in the uptake experiments. The electron binding energy (BE) of this species is 402.2(2) eV, referenced with the literature value of the O 1s BE of polycrystalline ice at 533.8 eV.^29^ Nitrogen species with a similar BE were previously observed in APXPS experiments of NO_2_ adsorbed on TiO_2_ and ascribed to reduced nitrogen.^30^ We refer to this nitrogen species in the following as N_adv_. This N_adv_ signature was invariant over an extended period indicating that it is not accumulating or subject to beam-induced effects (see ESI Fig. S2† for details).

The N 1s spectrum after adsorption of NH_3_ on the same ice substrate is shown in the upper trace of Fig. 2(a). It shows a significantly increased total intensity, with the strongest peak at the low BE side. Since this peak increases with increasing exposure to NH_3(g)_, we assign it to NH_3_ adsorbed on ice (blue shaded peak in Fig. 2(a)). The binding energy of adsorbed NH_3_, referenced to that of the O 1s peak of solid ice, is 400.7(2) eV, a value similar to that for NH_3_ adsorbed on silicon and silicate surfaces.^31,32^ The expected position of the gas-phase NH_3(g)_ peak, based on literature values (405.5(2) eV)^33^ that are referenced to a BE of water vapor (539.8(2) eV),^34^ is around 400 eV on the binding energy scale used in this study and depends on the degree of charging of the ice. The N 1s peak due to NH_3(g)_ thus overlaps with the signals of the adsorbates. However, due to the low partial pressure of NH_3(g)_ in the reactor cell the intensity of this peak is negligible within the signal-to-noise ratio in our experiments.

The additional signal at the high BE side of the spectrum (orange shaded peak) is more difficult to assign due to its overlap with the N_adv_ signature. This species could reasonably be interpreted as a time-dependent increase in the N_adv_ intensity, or that it is due to a new nitrogen species, for instance NH_4_^+^ which is formed by the protonation of adsorbed NH_3_. The latter would be consistent with observations in previous experiments.^15^

Since we cannot unambiguously assign this feature to NH_4_^+^, we label it for the moment as ΔN_adv,NH_4_^+^_. The ΔN_adv,NH_4_^+^_ peak has a binding energy of 402.5(2) eV, *i.e.* 1.8(2) eV higher than the BE of NH_3_, which is in good agreement with the value for NH_4_^+^ in aqueous solution.^35,36^ The higher binding energy of NH_4_^+^ compared to NH_3_ can be related to its positive charge. The sensitivity of XPS to the charge state has been used previously to discuss the protonation of acids at the solution–vapor^37^ and air–ice interface.^38–41^

### 3.2 Adsorption of NH_3_

We now turn our attention to the evolution of the N 1s spectra during the exposure of ice to NH_3(g)_ as a function of time and partial pressure. To follow and quantify the uptake of NH_3_ by ice, alternating N 1s and O 1s core level spectra were recorded using photon energies of 650 eV and 780 eV, respectively, to ensure a comparable probing depth, as described above. The N 1s spectra provide information on the chemical nature of the adsorbed NH_3_ species, while O 1s spectra serve as a reference to quantify the adsorbate concentration and to monitor potential charging effects due to the photoemission process.

Fig. 2(b) shows the experimentally-determined atomic N/O ratio for the ice film at −35 °C as a function of exposure time at two different nominal NH_3(g)_ partial pressures of *p*_1_ = 1.2 × 10^−3^ mbar and *p*_2_ = 4.3 × 10^−3^ mbar. The N 1s and O 1s intensities were normalized to the respective photoionization cross sections,^42^ and the photon flux. As the photoelectron intensity is directly proportional to the amount of the species of interest in the probed volume, the normalized N/O ratio serves as a measure of the concentration of adsorbed nitrogen species on ice. These data do not reveal the precise distribution of the adsorbates within the probed volume at the interface, *i.e.* whether they are just adsorbed to the surface or evenly distributed across the probed volume. For simplicity we present in Fig. 2(b) the volumetric concentrations assuming an even distribution of nitrogen species in the near-surface region.

The estimated volumetric nitrogen concentrations are shown on the right axis in Fig. 2(b), assuming one N atom per molecule. The red symbols represent the N/O ratio for the total nitrogen intensity, here referred to as N_total_, grey symbols show the N/O ratio of the reduced nitrogen N_adv_ determined from the deconvoluted N 1s spectra. Shaded background areas indicate the time intervals in which NH_3(g)_ was dosed onto the ice film. The N 1s spectrum for the freshly prepared ice film in the absence of NH_3(g)_ in the reactor cell is shown in Fig. 2(a), bottom trace, and was already discussed in the previous section. Upon adjusting the partial pressure to *p*(NH_3(g)_) = 1.2 × 10^−3^ mbar, the N/O ratio starts to increase. Over a time of around 100 min a rise in the N/O ratio is observed, with the N/O ratio roughly doubling over this time period. After about 100 min the N/O ratio reaches a plateau, indicating a steady state of NH_3_ adsorption/desorption.

When the flow of NH_3(g)_ into the reactor cell is stopped after about 250 min (see Fig. 2(b)), only a slight decrease in the N/O ratio is observed, most likely due to the slow pump-out of NH_3(g)_ from the reactor cell driven by retention and release from the reactor walls. Subsequently, at about 410 min the NH_3(g)_ partial pressure was increased to a higher value of *p*(NH_3(g)_) = 4.3 × 10^−3^ mbar, again resulting in a nonlinear increase in N_total_, eventually leading to a tripling of the original N/O ratio at about 500 min. The NH_3(g)_ flow was then stopped again, upon which a noticeable decrease of the N/O ratio is observed, indicating desorption of N species from the ice surface. A subset of the XPS spectra from which the N/O ratios in Fig. 2(b) are extracted is shown in the ESI.†

The fit of the N 1s data using constraints derived from the fit of the as-grown ice sample (Fig. 2(a)) shows that the peak area and thus the surface concentration of the adventitious N contamination (N_adv_), represented by the grey symbols in Fig. 2(b)), was constant during the whole time of the adsorption/desorption experiment. The increase in the N_total_ signal as a function of the exposure time is governed by the adsorption of NH_3_, which also shows reversibility under desorption conditions.

**Fig. 3:**
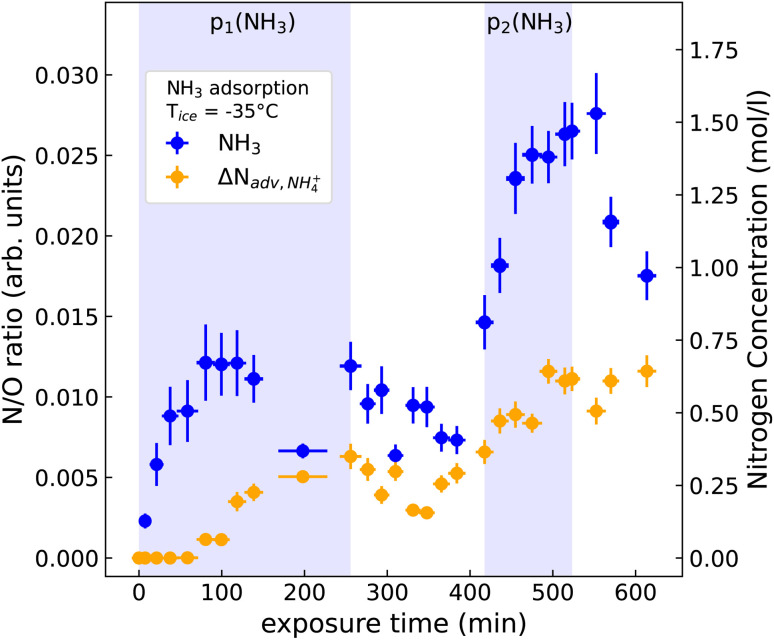
N/O ratio and estimated concentrations of NH_3_ (blue) and ΔN_adv,NH_4_^+^_ (orange) as a function of exposure time. Uncertainties on the *x*-axis stem from the acquisition times required for a set of N 1s and O 1s spectra. Uncertainties in the *y*-direction have been evaluated as the standard deviation (1*σ*) from the signal-to-noise ratios of the core-level spectra.

For a detailed look at the NH_3_ adsorption we plot the N/O ratios of the deconvoluted N 1s peak areas of NH_3_ (blue) and ΔN_adv,NH_4_^+^_ (orange) as a function of exposure time for the −35 °C ice sample in Fig. 3. The adventitious nitrogen contamination is not included in this graph. NH_3_ is the main adsorbed species and thus shows the same behavior with time and exposure as N_total_ in Fig. 2(b), *i.e.*, it displays an increase during NH_3(g)_ dosage and a noticeable decrease when the NH_3(g)_ flow into the reactor cell is stopped.

The ΔN_adv,NH_4_^+^_ species (see Fig. 3) shows a slightly different behavior compared to NH_3_ during NH_3(g)_ dosing, in particular a delayed appearance and slower increase in its abundance. This is observed for both NH_3(g)_ dosing steps, and also for the decrease in abundance when the NH_3(g)_ flow into the reactor cell is stopped. In particular in the case of desorption following the uptake of NH_3(g)_ at the higher partial pressure (*i.e.*, after 520 min in Fig. 3) the decrease in intensity of the ΔN_adv,NH_4_^+^_ peak does not follow that of the peak due to adsorbed NH_3_. Possible explanations for this behavior are: (i) that a fraction of the adsorbed NH_3_ undergoes protonation to NH_4_^+^, which has slower desorption kinetics; and (ii) that the amount of adventitious nitrogen (N_adv_) increases over time during the exposure to NH_3(g)_, possibly also due to photochemical reactions induced by the incident X-rays. While it is likely that NH_3_ engages in acid–base chemistry to form NH_4_^+^ as was observed in other studies,^15,18,43^ a change in N_adv_ cannot be completely ruled out due to the strong overlap in binding energy of the NH_4_^+^ and N_adv_ species.

### 3.3 Estimate of the NH_3_ concentration

The data presented above were obtained for an ice film at −35 °C. In addition, we have performed measurements at a number of other temperatures ranging from −23 °C to −52 °C, with each measurement starting with the preparation of a fresh ice sample. From the measured N/O ratios the concentrations of N_total_, N_adv_, NH_3_ and ΔN_adv,NH_4_^+^_ are determined and compiled in Table 1. The data show that *C*_N_adv__ increases with decreasing ice temperature. We note that the partial pressures of NH_3_, listed in Table 1, are upper estimates based on the concentration of gas entering the experimental set-up. Potential wall losses are not accounted for.

**Table 1:** Compilation of concentrations of nitrogen species on ice film between −23 °C and −52 °C. The concentrations have been determined from experimentally determined N/O ratios using XPS. *T*_ice_ is given in °C, *p*(NH_3_) in mbar and concentrations are given in mol l^−1^. Asterisks (*) indicate ice samples prepared in a separate experimental campaign in which N_adv_ was significantly higher

*T*_ice_	*p*(NH_3_)	*C*_N_total__	*C*_N_adv__	*C*_NH_3__	*C* (ΔN_adv,NH_4_^+^_)
−23	0.0043	1.9(3)	0.9(1)	0.5(1)	0.5(1)
−23	0.006	2.9(4)	0.9(1)	1.2(2)	0.8(1)
−29*	0.0012	7.3(1.0)	4.5(6)	1.8(3)	1.0(1)
−35	0.0012	2.1(2)	1.3(1)	0.5(1)	0.3(1)
−35	0.0043	3.3(3)	1.2(1)	1.5(1)	0.6(1)
−45*	0.0012	7.1(1.3)	5.5(1.0)	1.6(3)	0.0(1)
−45	0.0024	3.6(6)	2.1(3)	1.0(2)	0.5(1)
−45	0.006	4.4(6)	2.2(3)	1.7(2)	0.5(1)
−52	0.0012	5.8(1.1)	3.9(7)	0.7(1)	1.2(2)
−52	0.0024	5.3(7)	3.1(4)	1.3(2)	1.0(1)
−52	0.006	6.4(8)	3.1(4)	2.3(3)	0.9(1)

The clear separation of the NH_3_ species in the XPS spectra (Fig. 2(a)) allows us to discuss its concentration within our probing depth in more detail and set them in the context of literature values for the uptake of NH_3_ by ice and snow. The values for NH_3_ concentration from our measurements in Table 1 are of the order of 0.5 to 2.3 mol l^−1^ (*i.e.*, about 8 to 34 g l^−1^). These values are higher than the upper concentrations for ammonia or ammonium in bulk ice (up to 0.01 mol l^−1^ for NH_4_^+^).^19,44,45^ They are also higher than those found by Hoog and coworkers^18^ for ammonium trapped in bulk ice at −20 °C, which are <0.1 mg l^−1^ for similar gas-phase concentrations as in our experiments.

The higher concentration of NH_3(ads)_ in our experiments compared to the literature values obtain from volumetric measurements, indicates that NH_3_ is enriched in the surface region, since XPS exclusively probes the narrow interfacial region of the ice samples. If one assumes that all of the NH_3_ within our probing depth of about 1.7 nm is concentrated in a single layer at the very surface between the ice and vapor phase, the 2D concentration of NH_3_ would be about 3 × 10^14^ molecules per cm^2^, *i.e.* about one third of the concentration of water molecules in the surface layer. As we have already pointed out, we do not have information on the distribution of NH_3_ in the near surface layer, or the potential influence of the liquid-like layer, so the estimates for a pure surface layer (3 × 10^14^ molecules per cm^2^) and NH_3_ evenly distributed throughout the near-surface region (up to 2.3 mol l^−1^), are limiting cases for possible adsorption behavior scenarios. Either model shows, however, that the interfacial layer can hold even higher amounts of ammonium than the total amounts in the bulk of ice crystals. The fate of this interfacially trapped ammonium and ammonia over time needs further study to evaluate its impact on cloud scavenging.

## 4 Conclusions and outlook

In this article we showed that ambient pressure X-ray photoelectron spectroscopy is an excellent method to follow the uptake of ammonia on ice surfaces at atmospherically-relevant temperatures. We showed that APXPS is able to quantify the amount of adsorbed ammonia and to determine the chemical nature of the adsorbed species *via* the characteristic N 1s electron binding energy. The data demonstrate that ammonia adsorbs mainly in its neutral form (NH_3_), with some of the molecules most likely undergoing protonation to NH_4_^+^.

We were able to make these observation even in the presence of adventitious nitrogen contamination. This kind of contamination is a serious issue in any measurement under atmospherically-relevant conditions, *i.e.* far away from ultra-high vacuum conditions and at appreciable partial pressures of water vapor without large pumping speeds. This underlines the need for dedicated and easily cleanable reactor cells for studies of ice surfaces in the presence of reactive trace gases.

The present study builds on past experiments on the investigation of trace gas uptake by ice surfaces using APXPS.^29,38,46,47^ The success of these measurements opens up opportunities to not only study the adsorption of a single trace gas species, but also to investigate the co-adsorption and possible reactions of multiple trace gas species, with the ice surface potentially acting as a catalyst for a heterogeneous reaction between the adsorbates.

**Fig. 4:**
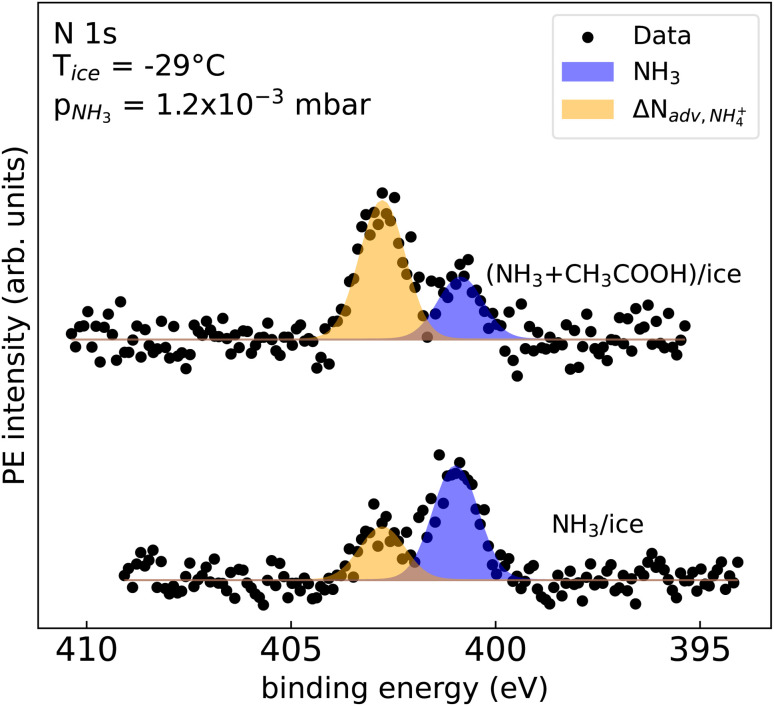
N 1s spectra of NH_3_/ice at −29 °C at a partial pressure *p*(NH_3_) of 1.2 × 10^−3^ mbar with (top) and without (bottom) acetic acid as the co-adsorbent. The spectra were scaled to the same background intensity. The linear background and the N_adv_ signal contribution was subtracted. The unsubtracted spectra are shown in the ESI.†

We therefore conclude this paper with the result of a proof-of-principle study of the co-adsorption of NH_3_ with acetic acid (CH_3_COOH). The N 1s spectra that compare the adsorption of NH_3_ with the case for CH_3_COOH/NH_3_ co-adsorption are shown in Fig. 4. The bottom trace shows the initially prepared NH_3_/ice surface at *p*(NH_3_) = 1.2 × 10^−3^ mbar. The top trace shows the same ice film after dosing NH_3_ and CH_3_COOH simultaneously. While the signal contribution of NH_3_ (blue) is more pronounced compared to ΔN_adv,NH_4_^+^_ (orange) on NH_3_/ice, the ΔN_adv,NH_4_^+^_ intensity significantly increases in the presence of CH_3_COOH. In addition a decrease in the NH_3_ intensity was observed. This indicates an interaction of NH_3_ and CH_3_COOH at the ice–vapor interface, likely leading to the formation of ammonium acetate.

We believe that this initial result holds promise for future investigations of more complex reactions at ice surfaces in the presence of a mix of trace gas species at their atmospheric concentrations and relevant ice temperatures. The strength of APXPS studies is that they are able to monitor the chemical nature of the adsorbate, *e.g.* its protonation state, and provide complementary information to flow tube studies, which are sensitive to the gas phase composition of reactants and products.

## Author contributions

CR conceived the project and analyzed the experimental data. CR, TBR and HB wrote the manuscript with critical feedback from all co-authors. CR, SG, YM, TB, LL, TBR and HB performed measurements. LA provided the endstation and support for the spectroscopic measurements. HB, MA and TBR supervised the project.

## Conflicts of interest

There are no conflicts to declare.

## Supplementary Material

FD-258-D4FD00169A-s001

## Data Availability

Data for this article, including the data displayed in Fig. 2–4 will be made available at our Zenodo repository “Uptake of Ammonia by Ice Surfaces at Atmospheric Temperatures” upon acceptance of the manuscript.
