# Supplementary material for: Uptake of ammonia by ice surfaces at atmospheric temperatures
Source: Faraday Discuss. 2024 Nov 16;258:532–45. doi: 10.1039/d4fd00169a (PMC11883511; doi:10.1039/d4fd00169a)
Supplement: FD-258-D4FD00169A-s001 [file FD-258-D4FD00169A-s001.pdf]

Supplementary Information (SI) for Faraday Discussions.
This journal is © The Royal Society of Chemistry 2024

Electronic Supplementary Information to "Uptake of Ammonia by Ice Surfaces at Atmospheric Temperatures"

Clemens Richter[1], Shirin Gholami[1], Yanisha Manoharan[2], Tillmann Buttersack[1], Luca Longetti[2], Luca Artiglia[2], Markus Ammann[2], Thorsten Bartels-Rausch[2], and Hendrik Bluhm[1]

[1]Fritz Haber Institute of the Max Planck Society, Faradayweg 4–6, 14195 Berlin, Germany
[2]Laboratory of Atmospheric Chemistry, Paul Scherrer Institute, CH-5232 Villigen PSI, Switzerland

Contents

1 Energy reference **2**

2 Adsorption-Desorption **4**

3 XPS fit model **5**

4 Co-adsorption of NH_3 and acetic acid **8**

1 Energy reference

The binding energy of the N 1s signals, specifically the signature of N_{adv} on the freshly prepared ice surface was determined relative to the O 1s state of the ice film. For this purpose pairs of O 1s and N 1s spectra of the freshly prepared ice films were recorded at the same photon energy of 650 eV. Fig. S1 shows representative core-level spectra of an ice film prepared at –45°C.

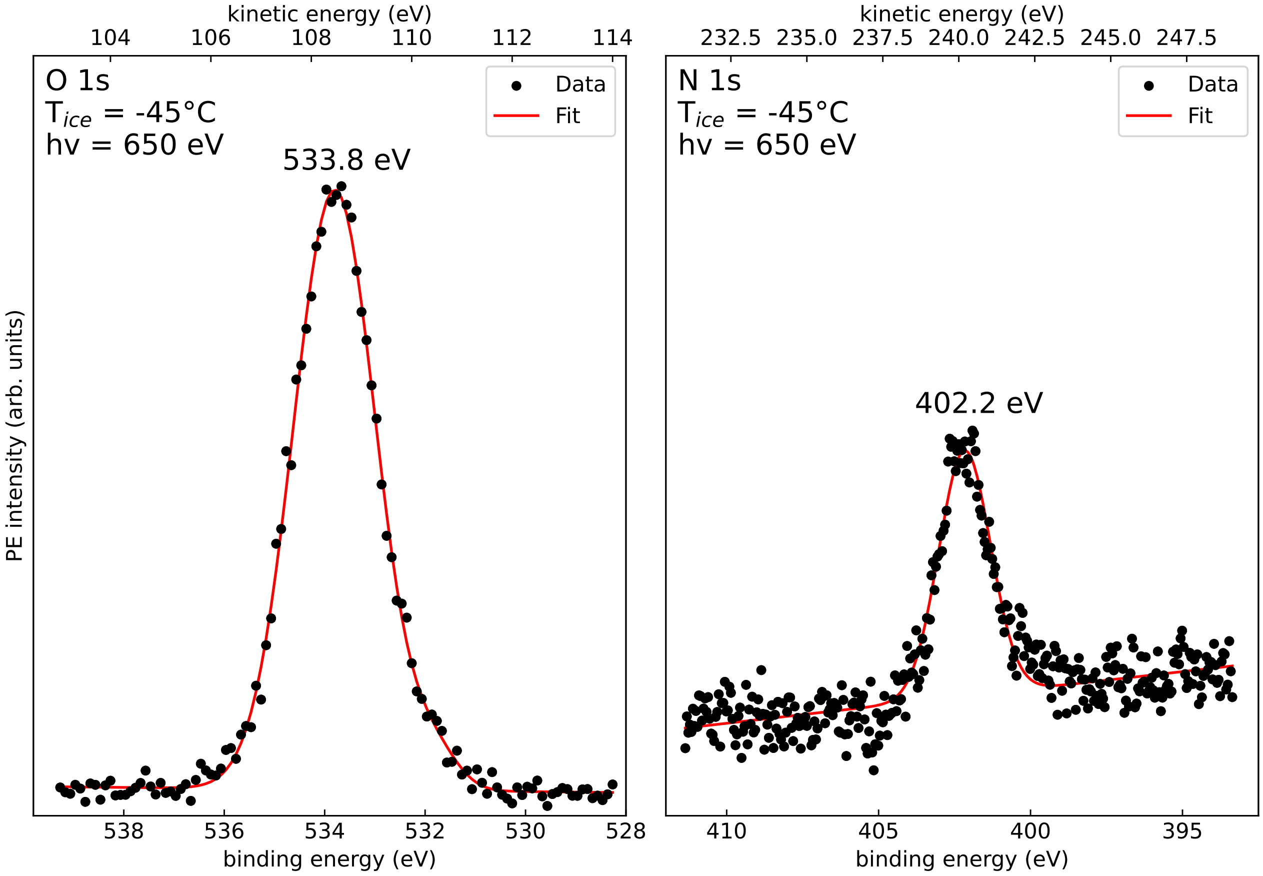

Figure S1: O 1s and N 1s spectra of freshly prepared –35°C ice at 650 eV.

The top axis shows the as-measured photoelectron kinetic energy, and the bottom axis the electron binding energy reference to the O 1s binding energy of water in clean ice at 533.8 eV.[2]. These measurements were performed for all ice films at –23 to –52°C; the binding energy of N_{adv} was consistently found to be 402.2(2) eV.

For each prepared ice film the cleanliness and stability was checked prior to NH_3 dosing by recording alternating O 1s and N 1s spectra. Here, 'stability' refers to a combination of two paramenters: (i) the establishment of a steady state of adsorption and desorption of the ice film and (ii) the stabilization of the X-ray-induced charging of the insulating ice film. The adsorption–desorption equilibrium determines the spatial stability of the ice surface with respect to the incident photon beam and the entrance aperture of the electron analyzer, and thus is importantant to ensure a stable photoelectron signal intensity as well as kinetic energy of the detected photoelectrons. The latter is influences by potential X-ray-induced charging. The degree of charging of the insulating ice film is dependent on the used photon energy and the total pressure and gas phase composition in the measurement cell.

In addition to determining the cleanliness and stability of the ice films, the temporal stability of the N_{adv} signature was tested. Fig. S2 shows representative N 1s spectra of a –45°C ice film prior to NH_3 dosing at three different times after initial film preparation.

The peak position in the spectra was aligned relative to the binding energy scale and the spectra normalized to the same background intensity to ensure their comparability. Over the course of approximately 8 hours no changes in the shape or intensity of N_{adv} were observed within the signal-to-noise level of our measurements. This observation allows to exclude the possible influence of beam damage on N_{adv} and the adsorption of further

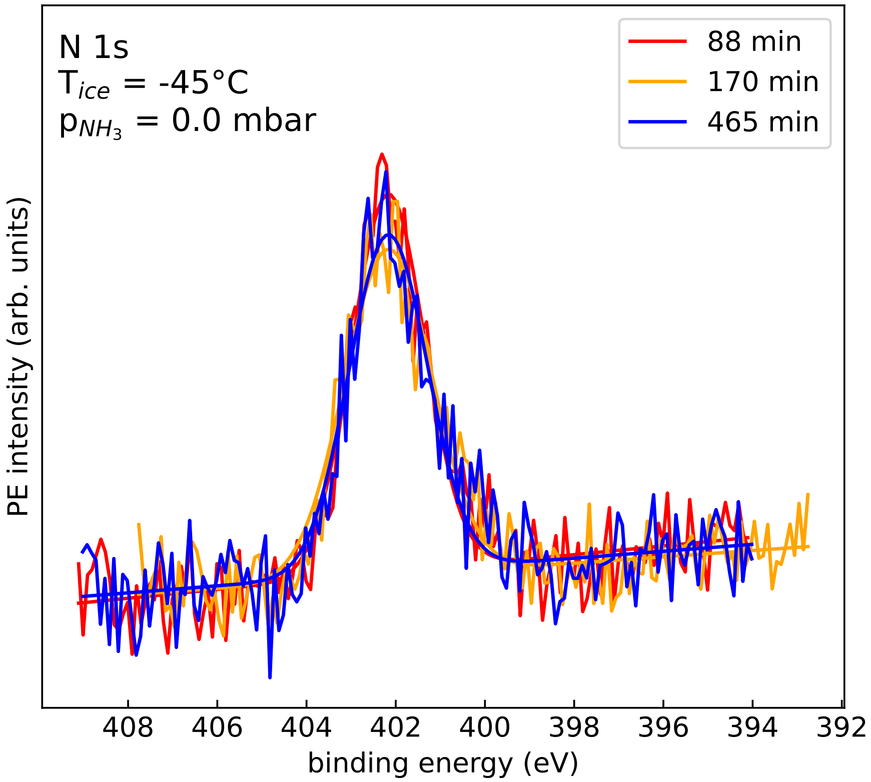

Figure S2: Evolution of N1s spectra of the freshly prepared ice film at at -45°C prior to NH_3 dosing. The experimental spectra are displayed in conjunction with the overall fit to guide the eye. All spectra have been normalized to the same background intensity to enable direct comparison of intensities.

N_{adv} contamination from the residual gas in the experimental cell over the course of the experiments.

2 Adsorption-Desorption

After successful confirmation of the stability of freshly prepared ice films, NH_3 was dosed onto the ice surface via a leak valve connected to a gas system, in which the partial pressure of NH_3 could be adjusted. The dosing process was monitored by alternating O 1s and N 1s spectra. Fig. S3(a) shows the evolution of the N 1s signal upon the adsorption and desorption of NH_3 on ice at –35°C and (b) the corresponding N_{total}/O ratio as a function of exposure .

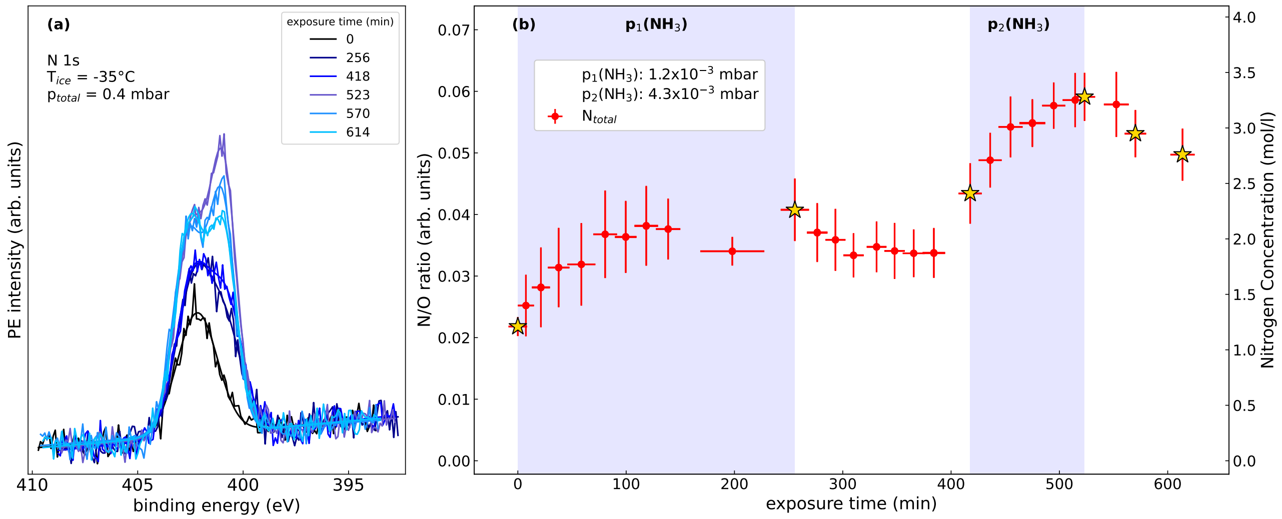

Figure S3: (a)Evolution of N1s spectra during the adsorption and desorption of NH_3 adsorbed on ice at –35°C. (b) Corresponding N_{total}/O ratio and estimated nitrogen concentration as a function of NH_3 exposure time. The shaded background area indicate the dosing of NH_3. White background indicates the absence of NH_3 flow into the experimental cell. The experimental spectra are displayed in conjunction with the overall fit to guide the eye. All spectra have been normalized to the same background intensity to enable direct comparison of intensities.

The spectra are aligned on the binding energy axis and normalized to the same background intensity. The color gradient indicates the respective order order the spectra from dark to light blue. In addition the temporal order of the acquisition of the spectra is indicated in Fig.S3(b) by star symbols. Fig.S3(b) shows that the overall N 1s intensity noticeably increases upon NH_3 exposure. The low binding energy side of the N 1s signal envelope shows the most prominent increase, correlating with the exposure of the ice surface to $NH_{3(g)}$. This correlation indicates that the low binding energy peak in the N 1s spectra is due to adsorbed NH_3.

3 XPS fit model

Due to the persistent presence of the N_{adv} signature in all spectra, a detailed discussion of the applied N 1s fit model is required. Based on the observations in Figs. S2 and S3(a), i.e., the temporal stability of the N_{adv} and the qualitative spectral changes upon NH_3 dosing, the simplest model to describe the N 1s spectra uses two fit components, one attributed to N_{adv} and the other to NH_3. Fig. S4 shows a representative fit using the two-component model for NH_3/ice at –35°C and $p_{NH_3} = 4.3x10^{-3}$ mbar.

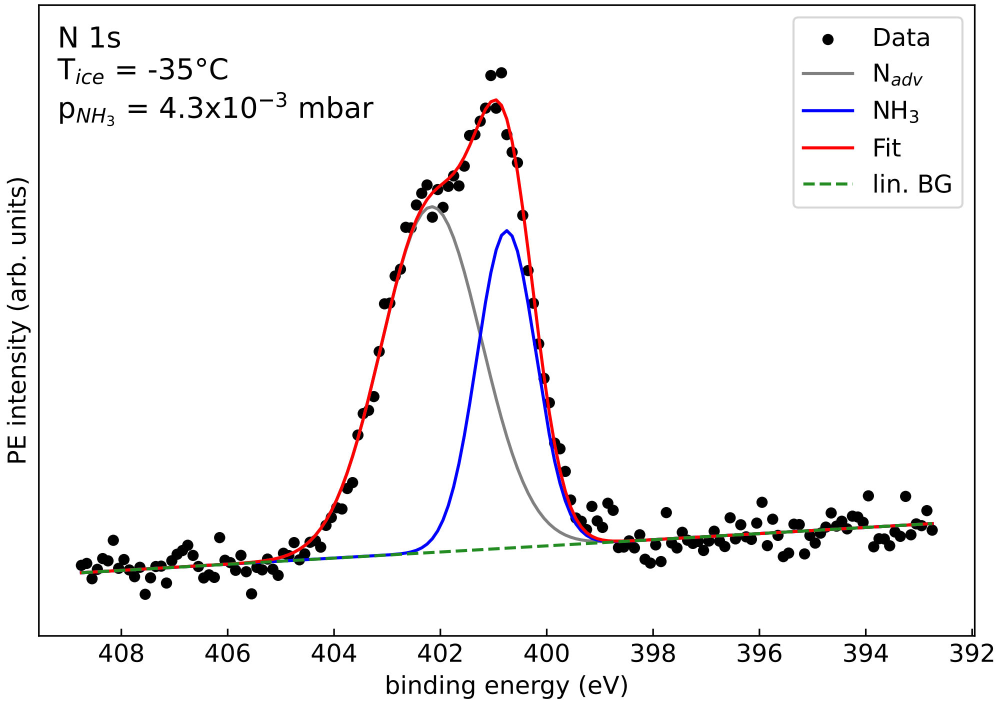

Figure S4: N 1s spectrum of –35°C NH_3/ice at 650 eV fitted using only two components (compare to Fig. S7).

Here, only the width of N_{adv} was constrained to the previously determined value for the freshly prepared ice film. The amplitude and position of N_{adv} as well as the NH_3 peak were free paramenters. The resulting optimized fit describes the experimental data reasonably well and allows the quantification of the NH_3 contribution to the N 1s spectrum. However, it consistently slightly overestimates the intensity around a binding energy of 404 eV and thus fails to capture the additional intensity observed in the spectra of co-adsorbed NH_3 and CH_3COOH on ice (see below in Fig. S8). Moreover, changes to N_{adv} are not immediately discernible. Therefore, a different fit model was used to evaluate the N 1s spectra of all prepared NH_3/ice films consisting of three contributions: (i) an invariant N_{adv} contribution, (ii) adsorbed NH_3, and (iii) an additional contribution describing adsorption-induced changes in the N_{adv} region denoted as $\Delta N_{adv,NH_4^+}$.

In the following the applied fit routine will be introduced. It was systematically applied to all data sets recorded for ice films over the temperature range between -23 °C to -52 °C. For each ice film, N 1s and O 1s spectra were recorded to characterize the initial state of the ice film. A representative N 1s spectrum obtained on a freshly-grown ice sample is shown in Fig. S5.

The spectrum is fitted using a single Gaussian distribution attributed to N_{adv} on a linear background. From this N 1s spectrum and accompanying O 1s spectra the N/O ratios were determined to estimate the concentration of N_{adv} on the ice film. Given the observed invariance of the N_{adv} signal (see Fig. S2), its width σ and amplitude in relation to the background intensity $\frac{A}{I_{lin.BG}}$ were set as constraints in the further analysis of the respective data set.

In a next step in the analysis the degree of X-ray-induced charging of the insulating ice film is estimated from O 1s spectra. Fig. S6 shows the O 1s spectra of the freshly-grown ice film and the NH_3/ice film at –35 °C at a function of the photoelectron kinetic energy.

The relative shift between the two spectra is defined as the change in X-ray-induced charging (Δq_{Xray}) and is used as a constraint in the further analysis of the NH_3/ice data set. Given the accuracy of 0.2 eV in the

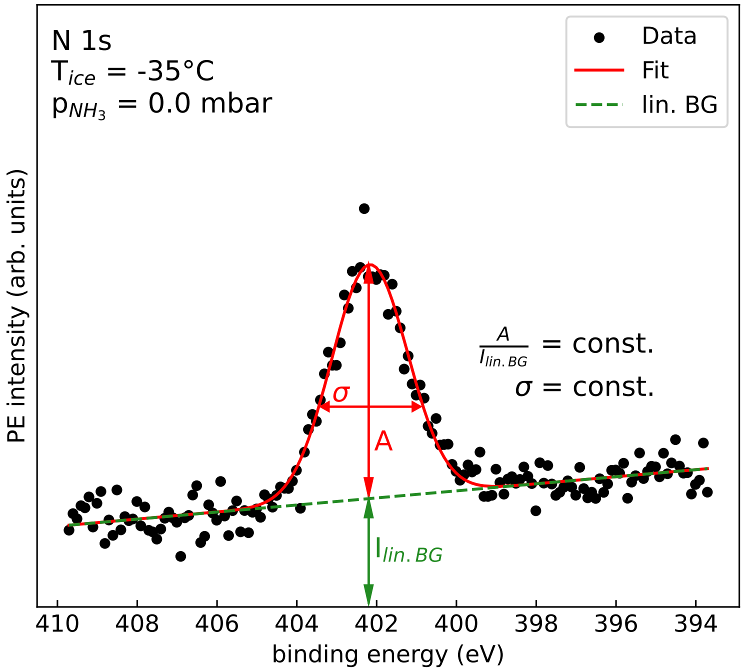

Figure S5: N 1s spectrum of freshly prepared ice at −35 °C ice, taken at an incident photon energy of 650 eV.

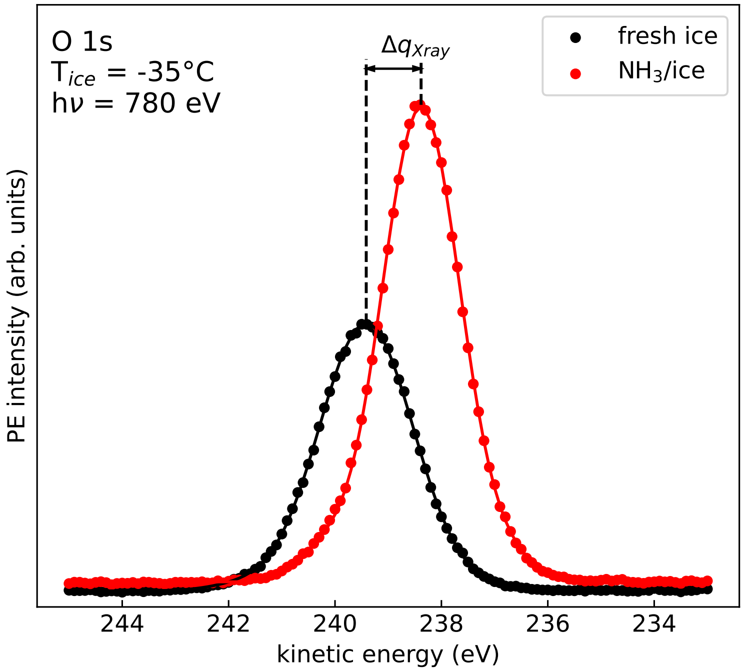

Figure S6: O 1s spectra of freshly-prepared ice at −35 °C ice and NH_3 adsorbed on ice, taken with a photon energy of 780 eV.

determination of O 1s and N 1s for the binding energy calibration, we estimate an accuracy of 0.4 eV for the respective value of Δq_{Xray}.

The N 1s spectra of NH_3 adsorbed on ice were fitted using the three peak components N_{adv}, NH_3, and $\Delta N_{adv,NH_4^+}$, with the constraints used for N_{adv} (with $\sigma_{N_{adv}}$ = const.). The other two peaks were described by Gaussians of equal width. A representative example of this fit model is displayed in Fig. S7.

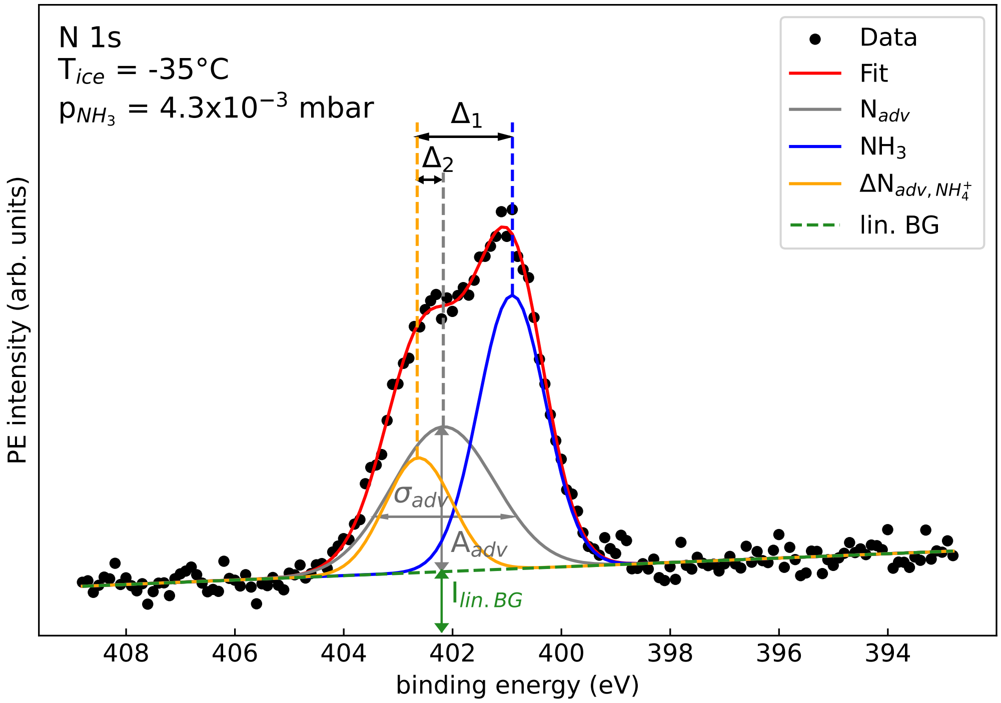

Figure S7: N 1s spectrum of NH_3 adsorbed on ice at −35 °C ice. The spectrum is fitted with 3 components (compare to Fig. S4).

For the evaluation of the two additional signal contributions one crucial assumption was made: NH_3 in aqueous environment is in equilibrium with its conjugate acid NH_4^+. Hence the notation $\Delta N_{adv,NH_4^+}$ for the third signal contribution as it is assumed that changes in the spectral region associated with N_{adv} could be attributed to the formation of NH_4^+. Therefore, the relative splitting Δ_1 between the two additional contributions was constrained within the ranges reported for NH_3 and NH_4^+ in aqueous solutions.[3, 4, 5, 1] The relative splitting Δ_1 between N_{adv} and $\Delta N_{adv,NH_4^+}$ was used as additional diagnostic parameter to test whether the fit results for N_{adv} and $\Delta N_{adv,NH_4^+}$ are within the energy ranges defined relative to the O 1s binding energy of 538.1(1) eV in liquid water.

The systematic application of this fit routine to all data sets resulted in the determination of binding energies of 400.7(2) eV for NH_3 and 402.5(2) eV for $\Delta N_{adv,NH_4^+}$ with respect to the O 1s binding energy of ice.[2] The presented values represent the average binding energies and standard deviation determined from all measurements summarized in Table 1 in the main text. The largest source of uncertainty is the position of N_{adv} due to X-ray-induced charging of the ice film; we estimate this to be of the order of 0.4 eV.

4 Co-adsorption of NH_3 and acetic acid

Co-adsorption experiments of NH_3 and acetic acid (CH_3COOH) were performed on a NH_3/ice film at −29 °C. Fig. S8 shows the N 1s spectra of NH_3/ice (bottom) and (NH_3+CH_3COOH)/ice (top), including the deconvolution of the individual signal contributions.

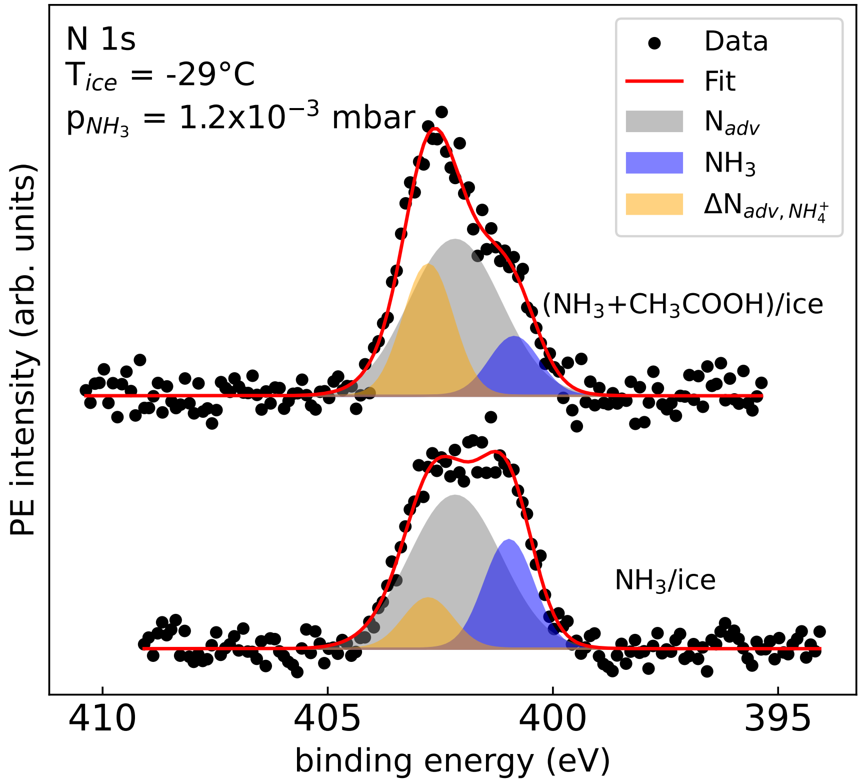

Figure S8: Deconvolution of N 1s spectra of NH_3/ice and (NH_3 + CH_3COOH)/ice, measured at -29 °C.

While for the case of NH_3 adsorbed on ice the signature of adsoebed NH_3 is more pronounced compared to $\Delta N_{adv,NH_4^+}$, their intensities are reversed in the presence of CH_3COOH. Since the acid-base reaction of NH_3 with CH_3COOH is an established route for the synthesis of ammonium acetate [NH_4][CH_3COO], the presence of NH_4^+ can be unambiguously shown to constitute a part of the $\Delta N_{adv,NH_4^+}$ signal.

References

[1] T. Gallo, G. Michailoudi, J. Valerio, L. Adriano, M. Heymann, J. Schulz, R. d. R. T. Marinho, F. Callefo, N. Walsh, and G. Öhrwall. Aqueous ammonium nitrate investigated using photoelectron spectroscopy of cylindrical and flat liquid jets. *The Journal of Physical Chemistry B*, 128(28):6866–6875, July 2024.

[2] A. Křepelová, J. Newberg, T. Huthwelker, H. Bluhm, and M. Ammann. The nature of nitrate at the ice surface studied by xps and nexafs. *Physical Chemistry Chemical Physics*, 12(31):8870, 2010.

[3] A. Lindblad, H. Bergersen, W. Pokapanich, M. Tchaplyguine, G. Öhrwall, and O. Björneholm. Charge delocalization dynamics of ammonia in different hydrogen bonding environments: free clusters and in liquid water solution. *Physical Chemistry Chemical Physics*, 11(11):1758, 2009.

[4] N. L. Prisle, N. Ottosson, G. Öhrwall, J. Söderström, M. Dal Maso, and O. Björneholm. Surface/bulk partitioning and acid/base speciation of aqueous decanoate: direct observations and atmospheric implications. *Atmospheric Chemistry and Physics*, 12(24):12227–12242, Dec. 2012.

[5] I. Unger, D. Hollas, R. Seidel, S. Thürmer, E. F. Aziz, P. Slavíček, and B. Winter. Control of x-ray induced electron and nuclear dynamics in ammonia and glycine aqueous solution via hydrogen bonding. *The Journal of Physical Chemistry B*, 119(33):10750–10759, Aug. 2015.
